# Programmable Electrostatics in Charge-Patterned Polypeptoid Micelles Probed by Small-Angle Neutron Scattering

**DOI:** 10.1021/acs.macromol.5c03433

**Published:** 2026-04-24

**Authors:** Erin Tsai, Chi-Huan Tung, Bailee N. Barrett, Guan-Rong Huang, Changwoo Do, Wei-Ren Chen, Donghui Zhang

**Affiliations:** † Department of Chemistry and Macromolecular Studies Group, 5779 Louisiana State University, Baton Rouge, Louisiana 70803, United States; ‡ Neutron Scattering Division, 6146 Oak Ridge National Laboratory, Oak Ridge, Tennessee 37831, United States; § Department of Engineering and System Science, National Tsing Hua University, Hsinchu 30013, Taiwan; ∥ Physics Division, National Center for Theoretical Sciences, Taipei 10617, Taiwan

## Abstract

Electrostatic interactions are key determinants in the assembly and organization of ionic micelles. Here, we synthesized sequence-defined polypeptoid block copolymers with discrete chain lengths and single or triple charged residues arranged in distinct patterns. Model-free small-angle neutron scattering analysis of semidilute polymer solutions at pH 9.0 revealed that intermicellar interactions follow screened Coulomb potentials, with strength and range determined by charge placement. Notably, positioning charged residues near the hydrophobic/hydrophilic block junction enhanced the range of electrostatic repulsion, while split-charge motifs produced stronger, longer-ranged repulsions than block-charge counterparts. Scattering length density profiles provide direct real-space insight into micellar dimension, core density, corona conformation, and solvent penetration. Micellar charge-to-aggregation number analysis further reveals the impact of invasive water and counterion association. This study demonstrates that sequence-specific charge patterning serves as a precise molecular tool for tuning micellar architecture and electrostatic interactions, establishing a foundation for programmable control of self-assembly in crowded environments.

## Graphical abstract

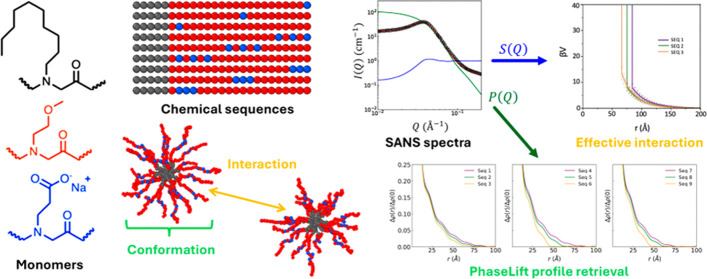

## Introduction

Biological macromolecules rely on molecular crowding, hydration, and finely tuned electrostatic interactions to control structure, dynamics, and assembly.^1–3^ In crowded environments where the average intermolecular distance is smaller than the molecular dimensions, crowding becomes a dominant force shaping biomolecular structure, dynamics, and function.^3–8^ While excluded volume effects and entropic constraints can stabilize compact conformations,^9,10^ accelerate protein folding,^11–13^ and modulate enzymatic activity,^14–16^ noncovalent interactions (both attractive and repulsive) may override these effects, destabilizing structures or promoting new modes of assembly.^17,18^ Moreover, compared to dilute environments, crowded conditions promote tighter hydration shell,^19^ reduce bulk water availability, and slow water diffusion dynamics.^19,20^ Collectively, these effects can stabilize compact biomolecular conformations, shift folding equilibria,^21^ and suppress non-native aggregation.^22^ Crowding, hydration, and electrostatics together shape biological organization at all scales.

Inspired by these biological principles, synthetic polymers have been studied in semidilute solutions, where interpenetration and crowding intensify steric and electrostatic constraints compared to dilute conditions.^23–25^ While dilute systems have yielded foundational insights into micellar self-assembly, they capture only a narrow window of self-assembly behavior. Above the overlap concentration, polymer chains interpenetrate,^23,24^ and crowding intensifies, giving rise to collective interactions that can reshape assembly pathways and resulting structures. Additionally, many practical micellar formulations operate well above the dilute regime, where intermicellar interactions become significant, such as in personal care products, food systems, and some drug delivery formulations. There is both fundamental interest and practical need to better understand intermolecular interactions and control polymer assembly in the semidilute regime. Earlier studies of surfactants, polyelectrolyte homopolymers, and charged block copolymers have demonstrated that charge density and concentration strongly affect micellar morphology and interparticle correlations.^26–35^ However, most synthetic systems lack molecular precision and cannot isolate sequence-level effects on electrostatics, leaving the role of spatial charge patterning under semidilute conditions poorly understood.^29,32,33^

Sequence-defined polypeptoids, in which side chains are appended to the polyglycine backbone nitrogen rather than the α-carbon, offer a versatile platform to address this challenge. Unlike polypeptides, polypeptoids lack backbone hydrogen bonding and stereogenic centers and enable precise monomer-by-monomer control over chemical sequence, side-chain functionality, and charge placement.^36–45^ This sequence programmability facilitates tuning of hydrophobic, hydrophilic, and charged residues, thereby allowing detailed control over chain conformation.^46–50^ Consequently, polypeptoids serve as ideal models for studying biomolecular charge encoding, while avoiding the conformational complexity inherent to polypeptides.^51–55^ Recent studies have shown that charge patterning in polypeptoids dictate chain conformation,^49^ micellar structure,^56,57^ intramicellar water distribution,^58^ pH-responsiveness^59^ and interfacial water dynamics of micelles.^60^ However, the interplay of charge number and spatial distribution along the polypeptoid chains under semidilute conditions-when micelles interact-remains largely unexplored.

Small-angle neutron scattering (SANS) provides quantitative access to both micellar form factors *P*(*Q*) and structure factors *S*(*Q*), thereby probing internal micellar structure and intermicellar interactions.^61–70^ However, traditional model-dependent approaches are limited in semidilute systems, where strong interparticle interactions distort scattering profiles and invalidate fixed-form models.^71–73^ To address this, recent advances have introduced model-free strategies including orthonormal basis expansion,^74^ PhaseLift reconstruction,^75^ and machine-learning inversion using Kolmogorov–Arnold networks (KAN).^76^ These approaches enable direct reconstruction of micellar density profiles and robust extraction of interparticle potentials from experimental data without restrictive assumptions.

In this work, we apply this model-free analysis framework to investigate the aqueous solution assemblies of sequence-defined polypeptoid block copolymers bearing one or three ionic monomeric residues strategically positioned within the hydrophilic block. By systematically varying the number and spatial arrangement of ionic residues (terminal, medial, and junctional placements; split versus block motifs), we uncover how charge encoding dictates the intramicellar structure and intermicellar electrostatic interactions under semidilute conditions. This work integrates model-free scattering analysis with molecularly programmed polymer design, utilizing charge patterning as a molecular handle to encode and control self-assembly in crowded environments, thus enabling the engineering of programmable, hierarchically organized soft materials with high precision.

## Materials and Methods

### Synthesis and Characterization of Sequence-Defined Polypeptoid Polymers

Sequence-defined polypeptoid block copolymers (Figure 1) were synthesized by the solid-phase submonomer method using an adapted procedure (Scheme S1) on a Prelude X Peptide Synthesizer.^36,41,56,58^ The resulting polymers have been characterized by reverse-phase high-performance liquid chromatography (RP-HPLC) and matrix-assisted laser desorption/ionization time-of-flight mass spectrometry (MALDI-TOF MS).

**Figure 1:**
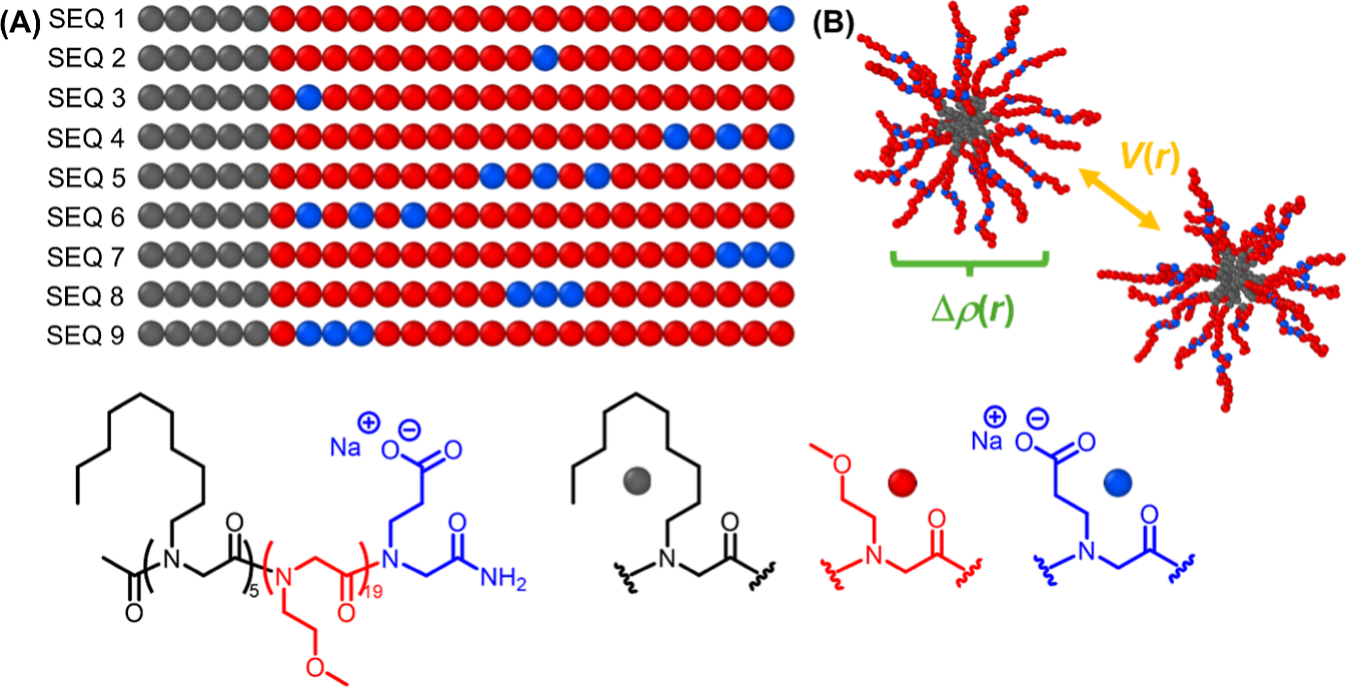
(A) The sequence library of polypeptoid block copolymers comprised of *N*-*n*-decylglycine (black sphere), *N*-2-methoxyethyl glycine (red sphere), and *N*-2-carboxyethylglycine residues (blue sphere), and chemical structure of a representative sequence-defined polypeptoid BCP (SEQ 1). (B) A conceptual illustration of hypothetical intramicellar structure and intermicellar interactions of sequence-defined polypeptoid BCP micelles (SEQ 5), to be probed by model-free SANS analysis. *V*(*r*) and Δρ­(*r*) denote the effective pair potential and excess scattering length density profiles, respectively.

### Reverse-Phase High Performance Liquid Chromatography

HPLC analysis was performed with a Waters 616 pump, Waters 2707 Autosampler, and a 996 Photodiode Assay Detector which are controlled by Waters Empower 2 Software. Separation was performed on a Waters XSelect HSS Cyano column (3.5 μm, 75 × 3 mm) by a gradient resulting from mixing eluents A (0.1% TFA in water) and B (0.1% TFA in acetonitrile). The gradient ran from 40% B to 70% B in 30 min. The flow rate was 0.4 mL/min, and the detected wavelength was 215 nm. Samples were prepared for analysis by dissolving the peptoid block copolymers (0.5 mg/mL) in a 60% water/40% acetonitrile-solvent mixture.

### Matrix-Assisted Laser Desorption/Ionization Time-of-Flight Mass Spectrometry

MALDI-TOF MS measurements were conducted on a Bruker UltrafleXtreme tandem time-of-flight (TOF) mass spectrometer equipped with a smartbeam-II 1000 Hz laser (Bruker Daltonics, Billerica, MA). The instrument was calibrated with Peptide Calibration Standard II (Bruker Daltonics, Billerica, MA). A saturated solution of α-cyano-4-hydroxycinnamic acid (CHCA) in methanol was used as the matrix in all measurements. The polymer solution samples (1 mg/mL) were mixed with the saturated matrix solutions at 1:1 volume ratio mixed thoroughly. The mixtures (1 μL) were deposited onto a 384-well ground-steel sample plate and were allowed to dry in air prior to measurement using positive reflector mode. Data analysis was carried out using FlexAnalysis software.

### Small-Angle Neutron Scattering Experiments

Aqueous polypeptoid BCP solutions were prepared by dissolving the lyophilized polymers in D_2_O at a concentration of 20 wt.%. Solutions were annealed at 70 °C for 2 h in a water bath and subsequently cooled to room temperature overnight (>8 h) to ensure equilibration. The pH of each solution was adjusted to 9.0 using NaOD (for D_2_O-based samples), ensuring full deprotonation of the ionizable groups. Ultrasmall-angle neutron scattering (USANS) measurements were carried out at the Spallation Neutron Source (SNS) at Oak Ridge National Laboratory. The samples were placed in quartz banjo cells with a path length of 2.0 mm. During data acquisition, the cells were gently tumbled in situ to prevent sedimentation and to maintain homogeneous exposure of the sample to the neutron beam. The incident neutron wavelength was set to 3.6 Å. The instrument configuration employed a source-to-sample distance of 30 m and an effective sample-to-detector distance of 2.13 m with a 1.2 cm pinhole aperture. This setup provided access to a scattering vector range from 1 × 10^–4^ to 1 × 10^–3^ Å^–1^. Data for each sample were collected over a measurement period of approximately 8 h, and the uncertainties in *I*(*Q*)­were determined based on counting statistics.

Small-angle neutron scattering (SANS) measurements were performed on the Extended *Q*-Range Small-Angle Neutron Scattering (EQ-SANS) instrument at the Spallation Neutron Source (SNS), Oak Ridge National Laboratory (ORNL).^77,78^ Solutions were loaded into 1 mm Hellma banjo cells and maintained at 25 °C during data acquisition. Scattering data were collected over a momentum transfer range of 0.007–0.2 Å^–1^ utilizing a sample-to-detector distance of 4.0 m in frame-skipping mode with a measurement time of approximately 30 min per sample. Raw scattering intensities were reduced to absolute units following standard EQ-SANS procedures, including corrections for detector sensitivity, transmission, and background subtraction.^79,80^ The measured absolute scattering intensity *I*(*Q*) is described by the standard factorization

$$I\left(Q\right)=n_{p}V^{2}{\left(\Delta \rho \right)}^{2}P\left(Q\right)S\left(Q\right)+I_{inc},$$

where *n*_p_ is the particle number density, *V*_p_ is the particle volume, Δρ is the scattering length density contrast, *P*(*Q*) is the single-particle form factor, *S*(*Q*) is the interparticle structure factor, and *I*_inc_ is the *Q*-independent incoherent background.

### Desmearing of *I*(*Q*)

The measured scattering intensities *I*(*Q*) were first corrected for instrumental resolution smearing prior to further analysis. This was accomplished using the central-moment-expansion method recently introduced by Huang and co-workers.^81^ Unlike conventional iterative or model-based desmearing approaches, this method achieves desmearing in a model-free manner, without requiring presumptive functional forms of the scattering cross section. The algorithm reconstructs resolution-free *I*(*Q*) directly from experimental data while preserving numerical stability and enabling reliable estimation of uncertainties. The desmeared *I*(*Q*) data sets were then used as input for subsequent decomposition into form factor *P*(*Q*) and structure factor *S*(*Q*).

## Results and Discussion

### Synthesis and Characterization of Sequence-Defined Polypeptoid Block Copolymer

A library of single or triple charged sequence-defined polypeptoid block copolymers (BCP) with discrete chain lengths and precise monomer sequence (Figure 1A) were synthesized by adapting a solid-phase submonomer synthesis method.^36,41,56,58^ Each polypeptoid BCP was composed of 25 total monomer residues (DP = 25). The hydrophobic block contained five *N*-*n*-decylglycine (*N*_DE_) residues, while the hydrophilic block contained twenty residues in total, consisting of neutral *N*-2-methoxyethyl glycine (*N*_ME_) residues and ionizable *N*-2-carboxyethylglycine (*N*_CE_) residues in varying number and spatial arrangement along the chain. For the single charged polypeptoid BCP (SEQ 1 – SEQ 3, Figure 1A), there are nineteen neutral *N*_ME_ residues and one ionizable *N*_CE_ residue in the hydrophilic block. The position of the single ionizable residue within the hydrophilic block was systematically varied, progressively moving it closer to the hydrophilic/hydrophobic block junction. Three distinct sequence-defined polypeptoid BCP (SEQ 1– SEQ 3) were thus prepared, representing terminal, medial and junctional placement of the ionizable residue, as illustrated in Figure 1A.

For the triple charged polypeptoid BCPs, there are seventeen neutral *N*_ME_ residues and three ionizable *N*_CE_ residues in the hydrophilic block (SEQ 4 – SEQ 9, Figure 1A). The three ionizable *N*_CE_ residues were arranged either in a block or split pattern and positioned progressively closer to the hydrophobic/hydrophilic block junction, similar to the approach used in the single charged polypeptoid BCP series. The molecular composition and purity level of each sequence was verified by high-performance liquid chromatography (HPLC) and matrix-assisted laser desorption/ionization time-of-flight mass spectrometry (MALDI-TOF MS) (Figures S1 and S2).

### Model-Free SANS Analysis of Semidilute Aqueous Polypeptoid Block Copolymer Solution

Small-angle neutron scattering (SANS) experiments were conducted to probe the intramicellar structure and intermicellar interactions of sequence-defined polypeptoid BCP (SEQ 1 – SEQ 9, Figure 1) in semidilute solutions. Figure 2 displays the measured absolute scattering intensity *I*(*Q*) for aqueous solutions of polypeptoid BCP (SEQ 1 – SEQ 9) at 20 wt.% in 100% D_2_O at 25 °C and pH 9.0 (black symbols), together with the corresponding fits (red lines) obtained from a model-free analysis protocol. The pH was adjusted to 9.0 to ensure complete ionization of the carboxylic acid groups on the polymer, given a typical p*K*_a_ of 5–6 for these peptoid sequences.^56,58^ For each sequence, the experimental profiles are decomposed into the single-particle form factor *P*(*Q*) (green curves) and the interparticle structure factor *S*(*Q*) (blue curves). The form factor *P*(*Q*) was extracted in a model-free manner using the orthonormal basis expansion method and PhaseLift algorithm,^74,75^ which reconstructs the excess scattering length density (SLD) distribution of micelles Δρ­(*r*) without assuming a particular particle geometry (vide infra). Δρ­(*r*) profiles offer direct real-space insight into the radial density distribution within individual micelles, revealing core density, coronal chain conformation, radial gradients, and solvent penetration. In parallel, the structure factor *S*(*Q*) was analyzed using a Kolmogorov–Arnold Network (KAN) approach,^76^ enabling extraction of the effective intermicellar interaction potential *V*(*r*) and its defining parameters, including interaction amplitude (*A*), Debye screening constant (κ), effective charge (*Z*), and hard-sphere diameter (*D*) (vide infra). Taken together, the agreement between the experimental scattering profiles and the model-free reconstructions (Figure 2) highlights the capability of this combined model-free framework to resolve both intra- and intermicellar structure in sequence-defined polypeptoid assemblies.

**Figure 2:**
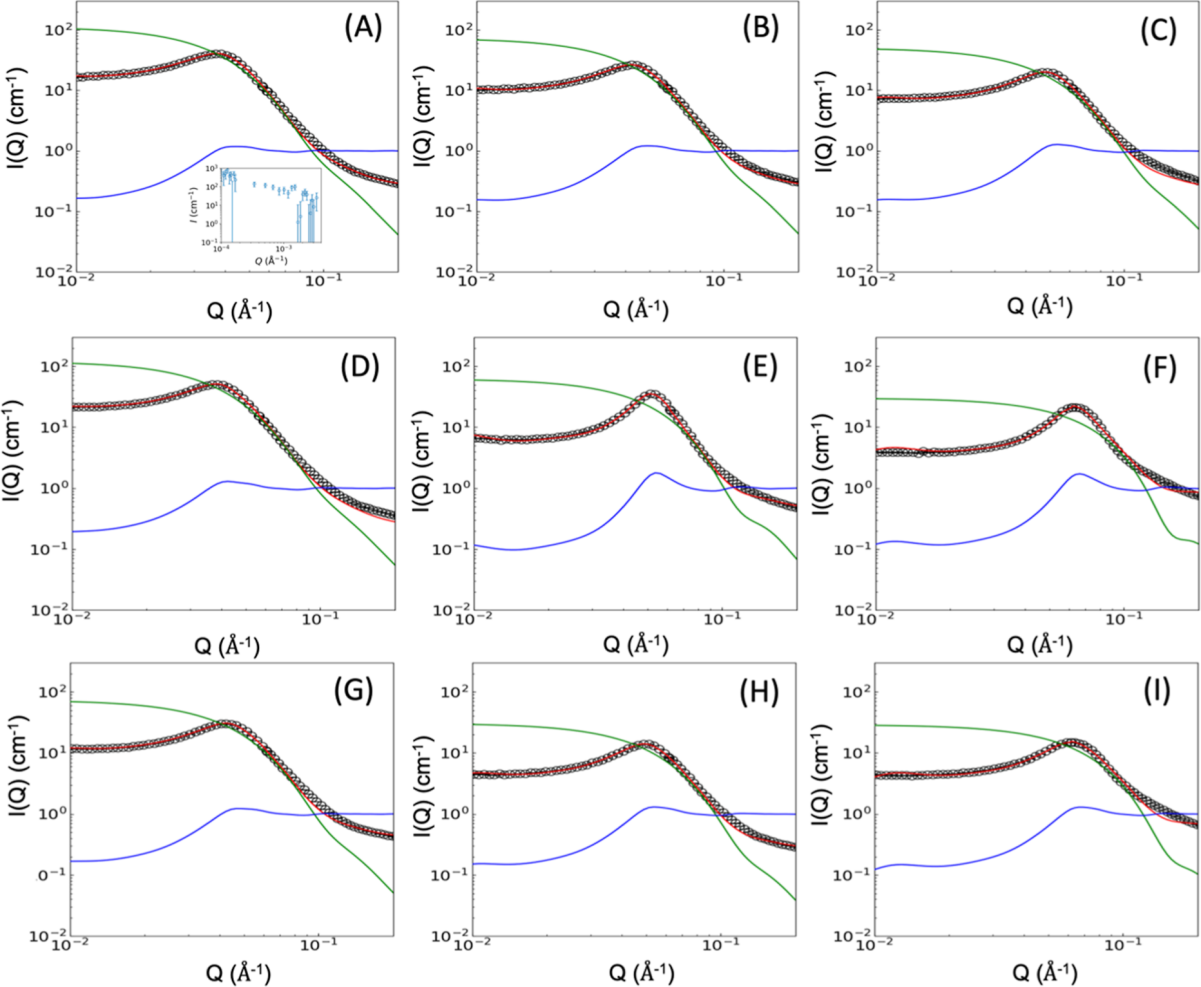
(A–I) Scattering intensity *I*(*Q*) for sequence-defined polypeptoid block copolymer micelles (SEQ 1 – SEQ 9) (black symbols) in semidilute aqueous solution (20 wt.% polymer in 100% D_2_O solution, pH = 9.0) with fits (red lines), decomposed into *P*(*Q*) (green line) and *S*(*Q*) (blue line) to extract interaction parameters and real-space SLD profiles Δρ­(*r*). An example of the USANS data is shown in the inset of panel (A), demonstrating the absence of detectable large-scale aggregation or mesoscale heterogeneity in the system.

The form factors (*P*(*Q*)) of SEQ 1 – SEQ 9 micelles exhibit three distinct regimes, as indicated by the green lines in Figure 2A–I. In all *P*(*Q*) profiles, the first regime is marked by a plateau in the low *Q* region (0.007 < *Q* < 0.02) in all *P*(*Q*) profiles. The second regime features a pronounced dip as *Q* increases within the intermediate *Q* range (0.02 < *Q* < 0.1). At higher *Q* values (*Q* > 0.1), all *P*(*Q*) profiles show a more gradual decay. For the single-charge sequences (SEQ 1 – SEQ 3), moving the ionic monomer closer to the junction of hydrophobic and hydrophilic blocks leads to a steady decrease of the plateau intensity in the low *Q* region and a more gradual decay in the intermediate *Q* region, indicating a consistent reduction in micellar aggregation number and size (Figure 2A–C). Similar trends are observed in the *P*(*Q*) profiles of the triple split-charge (SEQ 4 – SEQ 6, Figure 2D–F) and triple block-charge sequences (SEQ 7 – SEQ 9, Figure 2G–I).

The structure factor profiles (*S*(*Q*)) of SEQ 1 – SEQ 9 micelles each exhibited a distinct peak in the intermediate *Q* range (red lines, Figure 2), indicating repulsive interactions among the micelles in the semidilute solution. For the single-charge sequences (SEQ 1 – SEQ 3), the peak position consistently shifted to higher *Q* values, indicating a decrease in intermicellar spacing, as the ionic monomer was positioned progressively closer to the junction between the hydrophobic and hydrophilic blocks (Figure 2A–C). Triple split-charge sequences (SEQ 4 – SEQ 6) and triple block-charge sequences (SEQ 7 – SEQ 9) exhibited similar trends in peak positions within the *S*(*Q*) profiles of their respective sequence series.

To ensure that no coherent scattering arises from structures exceeding the micellar size, we further probed the micellar solutions using ultrasmall angle neutron scattering (USANS). As the example shown in the inset of Figure 2A across the probed *Q* range 10^–4^ – 10^–3^ Å^–1^, there is no systematic low-*Q* upturn or excess intensity beyond statistical fluctuations. This confirms the absence of larger-scale aggregates and establishes that the measured intensity is dominated by single-micelle scattering across the relevant length scales.

## Effective Intermicellar Interaction Potential of Polypeptoid BCP Micelles

For the ionic polymer micelles, the dominant pairwise intermicellar interaction arises from screened electrostatic repulsion, commonly represented by a hard-sphere Yukawa (HSY) potential derived from Debye–Hückel theory.^82,83^ This potential describes systems that have a hard-core repulsion at short range and a screened (Yukawa-type) repulsion at longer range, as follows

1 $$\beta V\left(r\right)=\{\begin{array}{l} \infty ,,r<D\\ A\frac{e^{-\kappa \left(r-D\right)}}{r},r\geq D \end{array}$$

where β (*=* 1/*k*_B_ *T*) is the Boltzmann factor, *D* is the hard-sphere diameter, κ $(=\frac{1}{\lambda_{D}}$ ) is the Debye screening constant (λ_*D*_ is the Debye length), and *A* (*=* $\frac{Z^{2}e^{2}}{{k_{B}T\varepsilon \left(1+\frac{\kappa D}{2}\right)}^{2}}$ ) is the interaction amplitude determined by the effective micellar charge number *Z*, solvent dielectric constant ε, and elementary charge *e.* Traditionally, the inversion of *S*(*Q*) to obtain *V*(*r*) has been performed using the Ornstein–Zernike (OZ) integral equation^61,84^ with closure approximations such as the mean spherical approximation (MSA),^63^ rescaled MSA (RMSA),^64^ and penetrating background RMSA (PB-RMSA),^70^ or the hypernetted chain (HNC)^66^ and Percus–Yevick (PY) closures.^67^ Each of these has well-documented limitations: HNC systematically underestimates S­(Q), PY overestimates it, RMSA and PB-RMSA break down at high concentrations, and the Rogers–Young (RY) closure,^68^ though effective at moderate coupling, remains computationally demanding and lacks robust convergence for strongly interacting systems.^69^ To overcome these limitations, we employed a machine learning-assisted framework based on the Kolmogorov–Arnold Network (KAN).^76^ This approach leverages the Kolmogorov–Arnold representation theorem to directly map scattering spectra onto effective interaction parameters. Unlike integral equation methods, KAN avoids assumptions about closure relations or analytic forms of the direct correlation function, providing a robust and generalizable regression scheme for extracting interparticle potentials from experimental *S*(*Q*).

Figure 3 displays the effective intermicellar interaction potentials, β*V*(*r*), reconstructed from the experimental *S*(*Q*) using the KAN-based inversion and fitted to a HSY form. For SEQ 1 – SEQ 3 (single charge), relocating the ionic monomer toward the hydrophilic/hydrophobic block junction increases both the contact repulsion and the range of the repulsive potential, consistent with more delocalized electrostatics as the charge approaches the micelle core. For SEQ 4 – SEQ 6 (triple charge, split) where the ionic residues are separated by neutral units, the inward relocation of charges increases the repulsive range of the contact potential. Compared to block-charge sequences at equivalent charge positions (SEQ 7 – SEQ 9), the split-charge sequences (SEQ 4 – SEQ 6) show stronger contact potential and more extended long-range repulsion, indicating greater charge delocalization along the hydrophilic block. SEQ 7 – SEQ 9 (triple charge, block), with clustered ionic residues, exhibit more localized interactions. As the ionic cluster move closer to the block junction, the decay range of β*V*(*r*) lengthens and the decay becomes markedly steeper compared to the split-charge counterparts.

**Figure 3:**
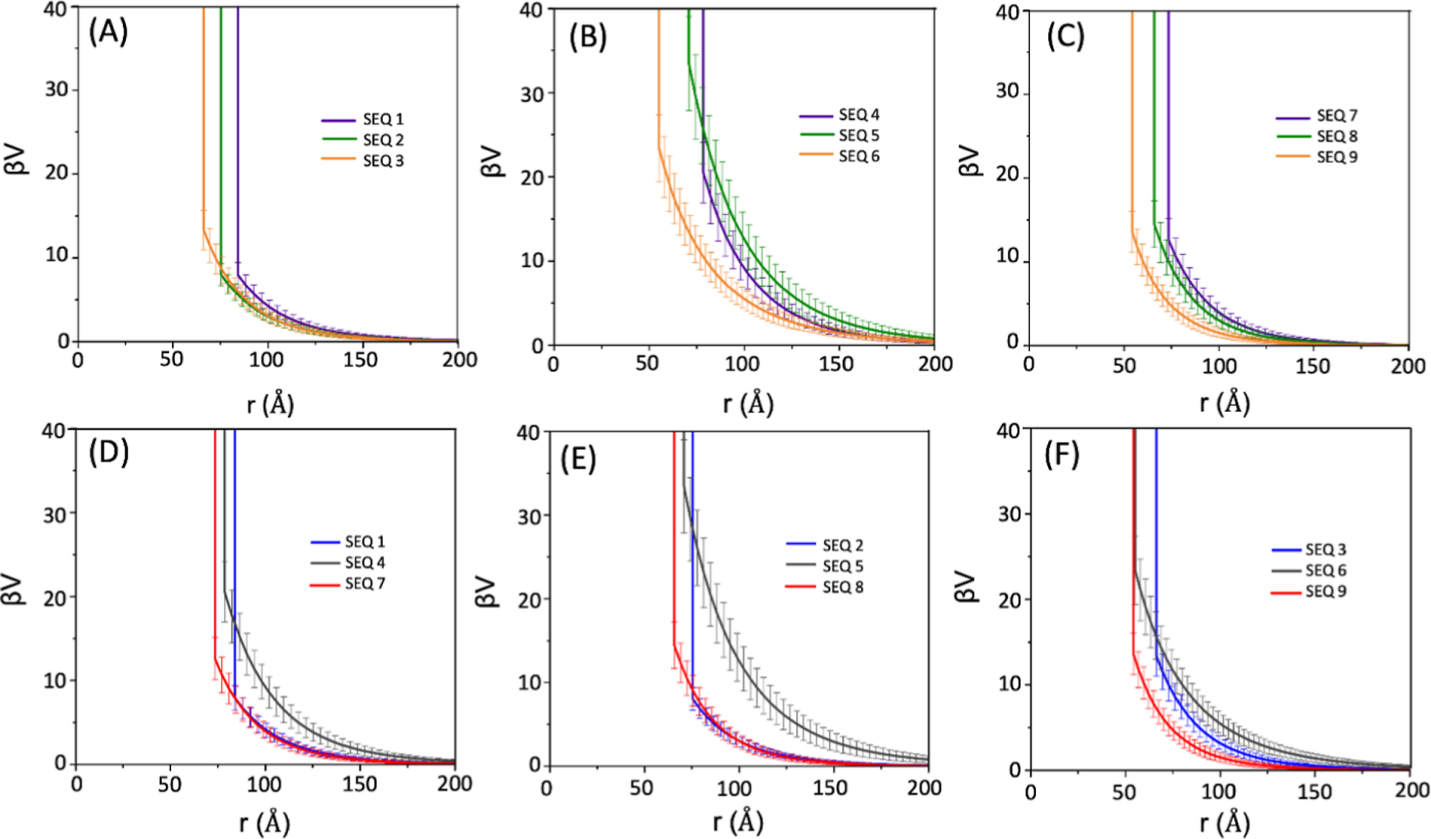
(A–F) Effective intermicellar interaction potentials β*V*(*r*) of the sequence-defined polypeptoid BCP micelles (SEQ 1 – SEQ 9) in semidilute aqueous solutions (pH = 9.0) obtained from HSY fits to *S*(*Q*) using the KAN-based inversion.

Cross-series overlays further emphasize that charge distribution, rather than charge number alone, determines the balance between repulsion strength and range (Figure 3D–F). Fitted HSY interaction potential parameters confirm these trends: inward positioning of charges along the chains decreases the hard-sphere diameter and increases the Debye screening length, indicating a more extended range of electrostatic repulsion (Table 1 and Figure 4). Furthermore, split-charge motifs consistently produce longer-ranged repulsion compared to block-charge counterparts.

**Table 1:** Intermicellar Interaction Potential Parameters for Sequence-Defined Polypeptoid Block Copolymer Micelles (SEQ 1 – SEQ 9) in Semidilute Aqueous Solutions (pH = 9.0)^a^ ^b^

sequence	in (*A*/*D*)	1/(κ*D*)	*D* (Å)	*Z*
	single charge (SEQ 1 – SEQ 3)			
1	2.1 ± 0.2	0.42 ± 0.04	83 ± 8	20 ± 2
2	2.1 ± 0.2	0.48 ± 0.05	74 ± 7	21 ± 2
3	2.7 ± 0.3	0.49 ± 0.05	65 ± 7	24 ± 2
	triple charge, split (SEQ 4 – SEQ 6)			
4	3.1 ± 0.3	0.50 ± 0.05	77 ± 8	35 ± 4
5	3.5 ± 0.4	0.67 ± 0.07	70 ± 7	54 ± 5
6	3.2 ± 0.3	1.0 ± 0.1	54 ± 5	51 ± 5
	triple charge, block (SEQ 7 – SEQ 9)			
7	2.6 ± 0.3	0.43 ± 0.04	73 ± 7	23 ± 2
8	2.7 ± 0.3	0.46 ± 0.05	65 ± 7	23 ± 2
9	2.7 ± 0.3	0.54 ± 0.05	54 ± 5	22 ± 2

a Values are reported with an estimated 10% uncertainty.

b The reduced interaction amplitude (ln (*A*/*D*)), reduced Debye screening length (1/(κ*D*)), hard-sphere diameter (*D*), and effective charge (*Z*) for SEQ 1 – SEQ 9 are summarized in Table 1 and Figure 4. ln­(*A*/*D*) reflects the contact potential, 1/(κ*D*) characterizes the spatial decay of electrostatic repulsion, *D* reflects the excluded volume of the micelles, and *Z* corresponds to the number of uncompensated charges per micelle inferred from the fits.

**Figure 4:**
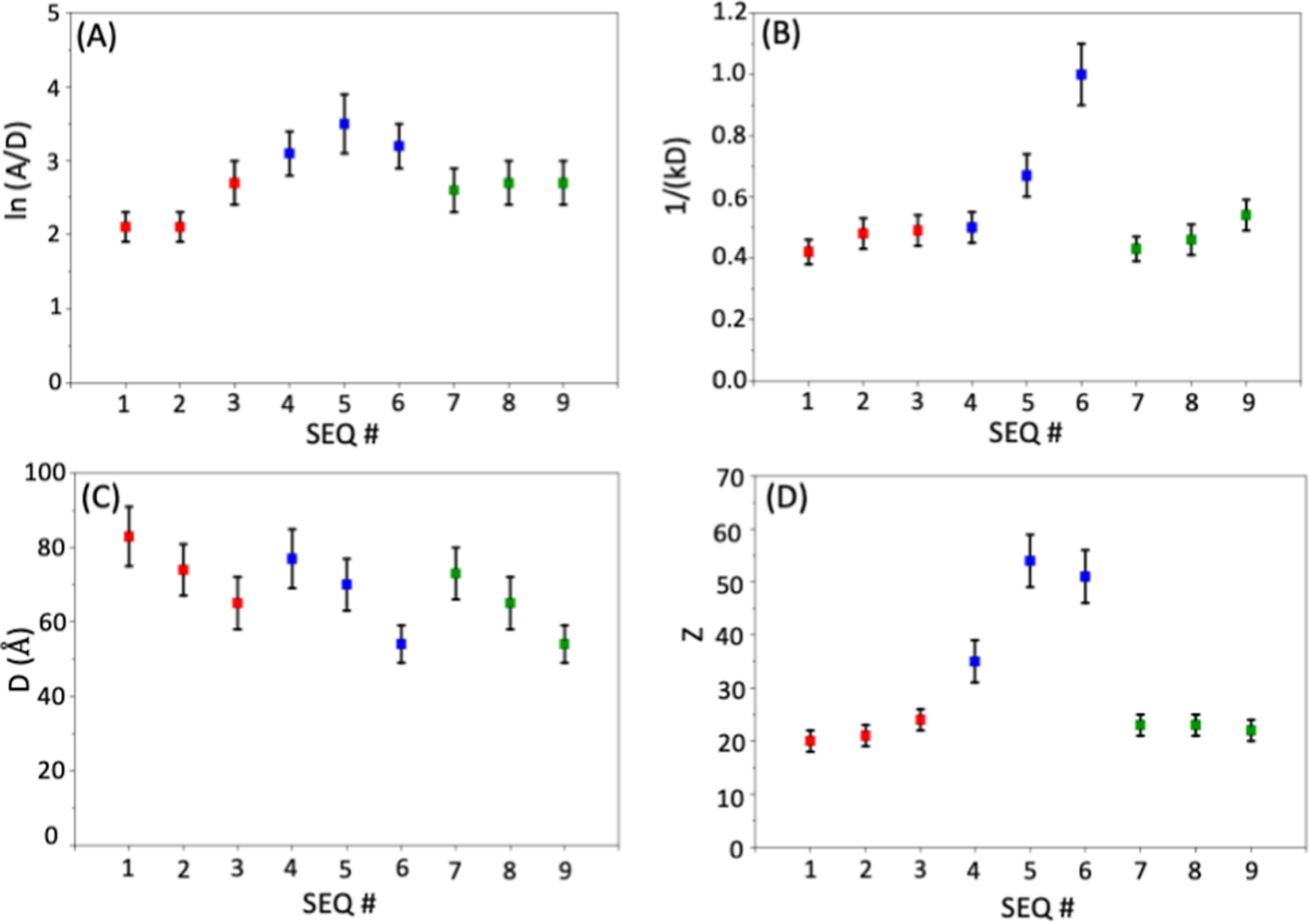
(A–D) Plot of effective interaction potential parameters for sequence-defined polypeptoid micelles (SEQ 1 – SEQ 9) in semidilute aqueous solutions (pH = 9.0). ln (*A*/*D*), 1/(κ*D*), (*D*), and *Z* correspond to the reduced interaction amplitude, reduced Debye screening length, hard-sphere diameter, and effective charge number of the micelles.

Within the single charge series (SEQ 1 – SEQ 3), relocating the ionic residue closer to the hydrophilic/hydrophobic block junction systematically decreases the *D* value, while increasing the ln (*A*/*D*), 1/(κ*D*), and *Z* (Figure 4). This indicates a smaller excluded micellar volume and stronger, longer-range electrostatic repulsion as the charge moves closer to the core-corona interface, reflecting more delocalized electrostatics. Practically, SEQ 1 exhibits the weakest, most localized repulsion, while SEQ 3 shows the strongest and most extended repulsion.

For the triple charge split sequences (SEQ 4 – SEQ 6), with ionic residues separated by neutral monomers along the hydrophilic block, ln (*A*/*D*) and *Z* values are substantially higher than in the single charge cases, reflecting the presence of multiple charges per chain (Figure 4A,D). These values are also significantly higher than those in the corresponding block-charge sequences (SEQ 7 – SEQ 9), indicating increased counterion association and consequently reduced contact potential in the latter (Figure 4A,D). Additionally, 1/(κ*D*) value is higher for the split-charge sequences, especially as charges approach the hydrophobic and hydrophilic block junction, signifying longer-ranged repulsion (Figure 4B). This suggests that charge delocalization extends the electrostatic field, enabling micelles to interact over greater distance, an effect amplified when the charges are positioned nearer the block junction.

To further examine the physical origin of this sequence dependence, it is instructive to consider the classical framework of Manning counterion condensation theory. Although Manning theory was originally developed for linear polyelectrolytes with a well-defined line charge density, the present system consists of globular micelles whose charged residues reside within a curved polymer corona. Therefore, a strict application of Manning theory to the micelle as a whole is not expected. Nevertheless, the Manning parameter provides a useful local electrostatic measure of charge spacing along the polymer backbone, which offers a physically meaningful consistency check for the observed trends.

Within Manning theory, counterion condensation occurs when the linear charge density parameter $\xi =\frac{l_{B}}{b}$ exceeds unity, where *l*_B_ is the Bjerrum length and *b* is the average spacing between neighboring charged sites along the chain contour. For aqueous solution at room temperature, the Bjerrum length is $l_{B}\approx 7Å$ . In polypeptoid chains, the projected backbone spacing between adjacent residues is approximately 3.5–4 Å. Consequently, for the block-charge motifs, where charged residues occur consecutively, the effective charge spacing is *b* ≈ 3.5–4 Å, giving ξ ≈ 1.75–2. In contrast, for the split-charge motifs, the charged residues are separated by neutral monomers, resulting in an effective spacing of *b* ≈ 7–8 Å, corresponding to ξ ≈ 0.88–1.

This comparison indicates that the block motifs locally exceed the Manning threshold (ξ > 1), which favors counterion condensation and reduces the effective charge observed at the micelle level. By contrast, the split motifs lie near or below this threshold, thereby reducing the degree of counterion condensation and yielding larger effective micellar charges *Z*. While the micelles themselves are not linear polyelectrolytes, this local charge-density argument provides a physically consistent explanation for why the split-charge sequences exhibit higher effective charges and longer-ranged electrostatic repulsion than the block-charge sequences.

Taken together, the fitted parameters show that electrostatic interactions are governed by both the total number and spatial distribution of ionic residues along the hydrophilic block. Split-charge sequences yield more delocalized, long-range repulsion, while block-charge sequences favor localized, short-range interactions. This underscores the central role of charge patterning in tuning the strength and spatial extent of intermicellar electrostatics.

### Intramicellar Structure of Polypeptoid BCP Micelles

While *S*(*Q*) encodes information about interparticle correlations, the form factor *P*(*Q*) reflects the internal structure of individual micelles. To obtain *P*(*Q*) and reconstruct the excess scattering length density profile Δρ­(*r*) in a model-free manner, we employed the orthonormal basis expansion and PhaseLift frameworks, which are mathematically equivalent.^74,75^ In these approaches, Δρ­(*r*) is expressed in terms of linearly independent basis functions subject to appropriate boundary conditions, and the corresponding scattering amplitude *F*(*Q*) and form factor *P*(*Q*) are calculated via spherical Bessel transforms.

This unified method eliminates the need for preassumed analytic models, which can bias interpretation and limit flexibility. Instead, *P*(*Q*) and Δρ­(*r*) (Figures S3–S6) are reconstructed directly from the measured *I*(*Q*), ensuring mathematical consistency between reciprocal and real space. Computational benchmarks and experimental demonstrations have confirmed that this framework provides stable and accurate solutions, even for diffuse or structurally complex micellar architectures.^75^ In our analysis, the extracted results obtained from the orthonormal basis expansion and PhaseLift were found to be in excellent quantitative agreement with one another, further validating the robustness of this model-free reconstruction. The most relevant outcome for quantifying intramicellar conformation is the reconstructed Δρ­(*r*) profile (Figures S3–S6), which represents a radially averaged measure of the internal density distribution in globular micelles. This profile collectively captures both the distribution of polymer segments and the penetration of water into the micellar interior, offering a comprehensive real-space description of micellar organization. The strong agreement between the two equivalent frameworks indicates that either method can be reliably applied in future studies of complex soft matter systems, providing a versatile and generalizable approach for scattering-based structural analysis.

Figure 5 shows the normalized excess scattering length density (SLD) profiles, Δρ­(*r*)/Δρ­(0), for SEQ 1 – SEQ 9 obtained using the orthonormal basis expansion and the described PhaseLift framework. All sequences exhibit core–shell-type micellar structures, as indicated by the presence of two distinct regimes in each Δρ­(*r*)/Δρ­(0) profile. In the first regime, corresponding to the micellar core region, Δρ­(*r*)/Δρ­(0) decreases sharply with increasing distance from the micellar origin (*r* = 0). In the second regime, associated with the corona domain, Δρ­(*r*)/Δρ­(0) decreases more gradually before asymptotically approaching zero (Figure 5). Notably, the lack of a plateau near the origin of the Δρ­(*r*)/Δρ­(0) profile suggests the presence of invasive water within the micellar core. For the single-charge series (SEQ 1 – SEQ 3), relocating the ionic residue closer to the hydrophilic/hydrophobic junction sharpens the decay of the Δρ­(*r*)/Δρ­(0) profile, indicating a more compact corona and enhanced localization of polymer segments near the core-corona interface (Figure 5A). This same trend in Δρ­(*r*)/Δρ­(0) is also observed for the triple charge split sequences (SEQ 4 – SEQ 6) and triple charge block sequences (SEQ 7 – SEQ 9), as shown in Figure 5B,C, respectively.

**Figure 5:**
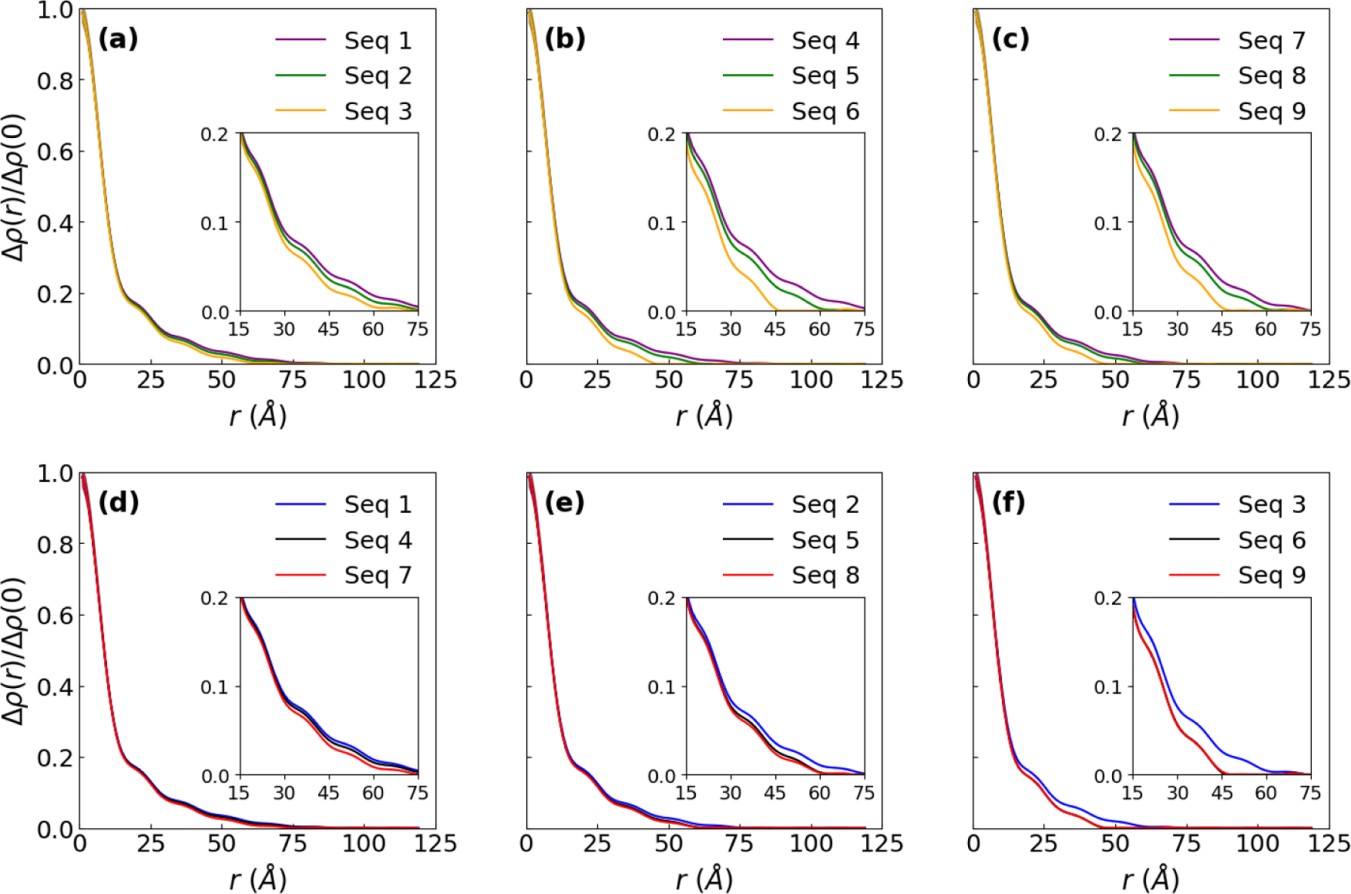
(A–F) Normalized excess scattering length density profiles Δρ­(*r*)/Δρ­(0), for sequence-defined polypeptoid block copolymer micelles (SEQ 1 – SEQ 9) in semidilute aqueous solutions (pH = 9.0), reconstructed using orthonormal basis expansion and PhaseLift frameworks. Error bars shown here represent the uncertainty from the orthonormal basis expansion and PhaseLift reconstruction and are smaller than the symbol size at the plotted scale.

Additionally, the triple-charge sequences exhibit a sharper radial decay in Δρ­(*r*)/Δρ­(0) compared to their single-charge counterparts with ionic residues positioned at equivalent locations along the chains (i.e., at terminal, medial or junctional position) (Figure 5D–F). This indicates that the triple-charge sequences form more compact micellar conformations with reduced radial extension. Furthermore, as shown in the inset of Figure 5D, SEQ 4 and SEQ 7 exhibit statistically significant differences in their Δρ­(*r*)/Δρ­(0) profiles. The triple split-charge sequence (SEQ 4), with charges positioned at the terminal ends of the chain, displays a broader and more intensified Δρ­(*r*)/Δρ­(0) profile across the radial distance range of 30Å < *r* < 75 Å. This suggests a delocalized charge effect and more extended coronas compared to the triple block-charge sequence (SEQ 7) (Figure 5D inset). Interestingly, when the charged monomers are located at the junctional and medial positions along the chain, the differences in the Δρ­(*r*)/Δρ­(0) profiles between the triple split-charge and triple block-charge sequences are greatly reduced (SEQ 5 vs SEQ 8, Figure 5E) or nearly eliminated (SEQ 6 vs SEQ 9, Figure 5F).

Overall, the reconstructed profiles demonstrate that Δρ­(*r*)/Δρ­(0) reflects both the intramicellar polymer distribution and the distribution of penetrated water. The systematic differences among SEQ 1 – SEQ 9 highlight that both the number of ionic residues and their spatial arrangement govern the micellar density organization in semidilute solution. From the reconstructed modified excess SLD profiles Δρ­(*r*) (*n*_m_)^1/2^ (*n*_m_ = micellar number density) shown in Figure S6, the radius of gyration *R*_g_, which characterizes the density-weighted spatial extent of the micelle, is defined as

2 $$R_{g}^{2}=\frac{\int_{0}^{\infty }r^{2}\Delta \rho \left(r\right)4\pi r^{2}dr}{\int_{0}^{\infty }\Delta \rho \left(r\right)4\pi r^{2}dr}$$

in addition, an effective micellar radius (*R*) can be introduced as

3 $$\frac{4\pi }{3}R^{3}=\frac{1}{\Delta \rho \left(0\right)}\int_{0}^{\infty }r^{2}\Delta \rho \left(r\right)4\pi r^{2}dr$$

where Δρ(0) denotes the central excess SLD, eq 3 defines an effective spherical boundary within which the density fluctuation is assumed homogeneous at the central contrast value. In the micellar context, if the core is assumed to be water-free, *R* provides a measure of the surfactant content per micelle (see eq 5 below), while *R*_g_ characterizes the spatial distribution and extent of the scattering density. Taken together, *R* and *R*_g_ provide complementary real-space descriptors of micellar size and internal organization. For a hard sphere, the ratio $\sqrt{5R_{g}^{2}/3R^{2}}$ = 1. For a general monotonically decreasing profile Δρ­(*r*), the ratio $\sqrt{5R_{g}^{2}/3R^{2}}>1$ reflects the broader spatial distribution of contrast beyond the effective homogeneous radius and serving as an indicator of the degree of close packing of the polymer contents in the micelles. In the context of micelles, a greater deviation of the ratio from unity ( $\sqrt{5R_{g}^{2}/3R^{2}}$ > 1) indicates a more diffused structure with larger radial extension. The values of *R*_g_, *R,* and $\sqrt{5R_{g}^{2}/3R^{2}}$ presented in Table 2 and Figure 6 corroborate the observed trend in the micellar structure reflected in the Δρ­(*r*)/Δρ­(0) profiles (Figure 5).

**Table 2:** Structural Parameters for Sequence-Defined Polypeptoid Micelles (SEQ 1 – SEQ 9) Obtained From Their Respective Modified SLD Profile Δρ­(*r*) (*n*_m_)^1/2^ (Figure S6) and eqs 2, 3 and 5

sequence	*R* (Å)	*R*_*g*_ (Å)	$\sqrt{5R_{g}^{2}/3R^{2}}$	*N*_ag*g*_	*Z*/(*N*_agg_ *n*_c_)
	single charge (SEQ 1 – SEQ 3; *n*_c_ = 1)				
1	25.0 ± 0.2	48.0 ± 0.5	2.48 ± 0.03	12.7 ± 0.4	1.6 ± 0.2
2	23.2 ± 0.2	42.6 ± 1.0	2.37 ± 0.06	10.1 ± 0.3	2.0 ± 0.2
3	21.3 ± 0.2	37 ± 1	2.26 ± 0.08	7.8 ± 0.2	3.1 ± 0.3
	triple charge, split (SEQ 4 – SEQ 6; *n*_c_ = 3)				
4	24.1 ± 0.2	45.9 ± 0.6	2.46 ± 0.04	11.9 ± 0.4	1.0 ± 0.1
5	20.9 ± 0.2	34 ± 2	2.1 ± 0.1	7.7 ± 0.3	2.3 ± 0.3
6	17.3 ± 0.2	26 ± 3	1.9 ± 0.2	4.4 ± 0.2	3.9 ± 0.4
	triple charge, block (SEQ 7 – SEQ 9; *n*_c_ = 3)				
7	22.6 ± 0.2	40.8 ± 0.8	2.33 ± 0.05	9.8 ± 0.3	0.8 ± 0.1
8	20.6 ± 0.2	35 ± 1	2.17 ± 0.09	7.4 ± 0.2	1.1 ± 0.1
9	17.5 ± 0.2	26 ± 3	1.9 ± 0.2	4.5 ± 0.2	1.6 ± 0.1

**Table 3:** Summary of Model-Free Analysis Framework

method	experimental input	intermediate quantity	extracted output	derived parameters
orthonormal basis expansion	*I*(*Q*)	*F*(*Q*),*P*(*Q*)	Δρ	*R*_*g*_,*R*,*N*_agg_
Phaselift	*I*(*Q*)	Δρ(*r*)Δρ(*r*)^*T*^	optimized Δρ	structural consistency check
KAN	*I*(*Q*)	direct correlation function	*V*(*r*)	κ,*D*,*Z*,*A*

**Figure 6:**
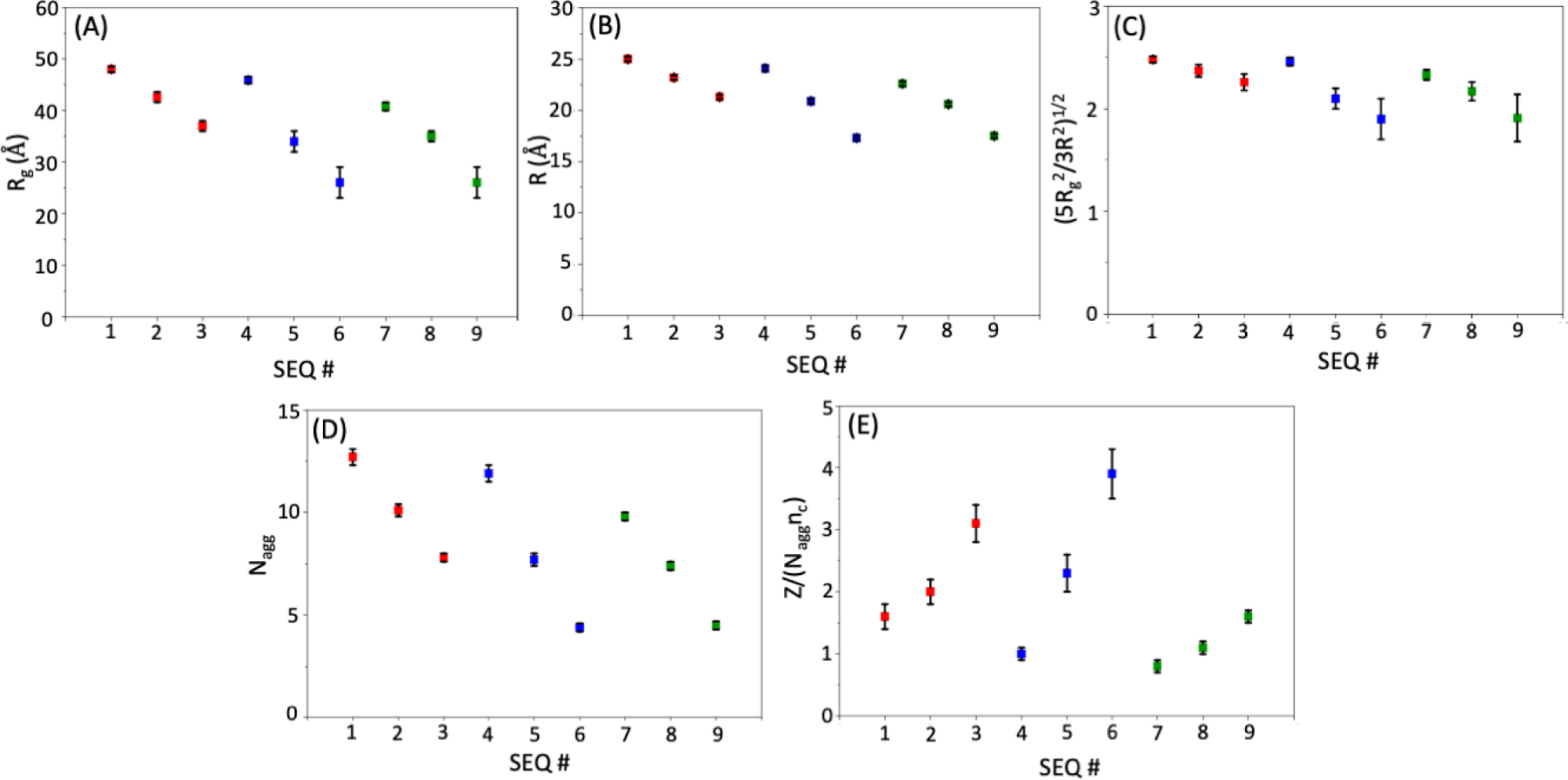
(A–E) Plots of structural parameters for sequence-defined polypeptoid micelles (SEQ 1 – SEQ 9) obtained from their respective modified SLD profiles Δρ­(*r*) (*n*_m_)^1/2^ (Figure S6) and eqs. 2, 3, and 5.

The micellar aggregation number (*N*_agg_) is defined as

4 $$N_{agg}=\frac{V_{m}}{v_{m}}=\frac{1}{{\Delta \rho }_{m}v_{m}}\int_{0}^{\infty }\Delta \rho \left(r\right)4\pi r^{2}dr$$

where *V*_m_ is the volume within a single micelle occupied by polymer chains, *v*_m_ is the molecular volume of a polymer chain, and Δρ_m_ is the difference in scattering length density between polymers and bulk water. If we assume the absence of invasive water at the micellar center, Δρ(0) = Δρ_m_. In this asymptotic scenario, *N*_agg_ can be obtained from the modified excess SLD profiles of the micelles (Figure S6) using eq 5, as summarized in Table 2.

5 $$N_{agg}=\frac{1}{{\Delta \rho }_{\left(0\right)}v_{m}}\int_{0}^{\infty }\Delta \rho \left(r\right)4\pi r^{2}dr$$

It is instructive to compare the effective charge *Z* (Table 1) with *N*_agg_ *n*_c_ where *N*_agg_ is the micellar aggregation number and *n*_c_ is the number of structural charges on a polymer chain (*n*_c_ = 1 for SEQ 1 – SEQ 3; *n*_c_ = 3 for SEQ 4 – SEQ 9) (Table 2). In principle, under the assumption of no invasive water in the micellar core, the relation of *Z* ≤ *N*_agg_ *n*_c_ is expected, reflecting varying extent of counterion association. Yet, the comparison shows that this relation is not always satisfied, as seen in Table 2. In view of eqs 4 and 5, this outcome underscores the influence of invasive water within the micellar core, which alters the excess scattering length density distribution, leading to an underestimation of *N*_*agg*_ and consequently altering the relationship between *Z* and *N*_agg_ *n*_c_.

Although the present SANS analysis cannot decouple the effect of counterion association and invasive water on Δρ­(*r*), the ratio *Z*/(*N*_agg_ *n*_c_) (Table 2) serves a useful collective indicator of both counterion association and invasive water penetration. It should be emphasized that the ratio *Z*/(*N*_agg_ *n*_c_)­is not interpreted here as a direct quantitative measure of solvent fraction within the micellar core. The aggregation number *N*_agg_ is estimated under the asymptotic dry-core assumption in eq 5, which provides an approximate measure of the polymer content per micelle. If invasive water is present within the micellar core, the reconstructed Δρ­(*r*) profile will underestimate the true polymer volume, leading to a systematic underestimation of *N*_agg_. Importantly, however, this bias affects the absolute magnitude of *N*_agg_ but not the relative comparison across sequences, since all samples are analyzed using the same contrast conditions and reconstruction protocol. Therefore, *Z*/(*N*_agg_ *n*_c_) should be interpreted as a relative indicator reflecting the combined effects of counterion association and invasive water penetration, rather than a direct quantitative determination of core hydration. Note that a *Z*/(*N*_agg_ *n*_c_) ratio greater than unity indicates a greater extent of invasive water within the micellar core, while a ratio less than unity does not necessarily signify the absence of invasive water. Based on the *Z*/(*N*_*agg*_ *n*_c_) values in Table 2, all micelles contain a significant amount of invasive water within the core, and the extent of invasive water increases with the inward placement of ionic residues for both single-charge and triple-charge, split or block sequence series (Figure 6E). Triple, split charge sequences (SEQ 4 – SEQ 6) show more invasive water in the micellar core than the triple, block charge counterparts (SEQ 7 – SEQ 9).

## Methodological Assumptions and Scope of the Model-Free Framework

Although described as model-free, the present analysis relies on well-defined mathematical constraints rather than predefined structural ansatz. The orthonormal basis expansion assumes that the excess scattering length density profile Δρ­(*r*) can be expressed as a linear combination of complete orthonormal basis functions satisfying physical boundary conditions, including finite radius of gyration and smooth decay at large *r*. No geometric form (e.g., core–shell or fuzzy sphere) is imposed. The PhaseLift reconstruction reformulates the inversion problem in a lifted matrix space *X* = ΔρΔρ*^T^*, converting the nonconvex phase retrieval problem into a convex optimization problem. This guarantees convergence to a global minimum under statistical isotropy and finite-support assumptions. The KAN-based inversion assumes only the existence of a continuous mapping between scattering observables and effective interaction parameters. Unlike Ornstein–Zernike integral equation approaches, it does not assume closure relations or analytic forms for the direct correlation function. These constraints differ fundamentally from traditional model-based fitting approaches, which presuppose explicit structural forms. Here, structural information emerges directly from the experimental data subject only to mathematical regularity and physical boundary conditions. Table 3 summarizes the data flow from experimentally measured *I*(*Q*) to real-space structural and interaction parameters without reliance on parametric structural models.

## Conclusions

In this work, we investigated the intra- and intermicellar structures of sequence-defined ionic polypeptoid block copolymers using a model-free small-angle neutron scattering (SANS) analysis framework. By integrating orthonormal basis expansion and PhaseLift reconstruction for single-particle form factor analysis, and Kolmogorov–Arnold networks (KAN) for structure factor analysis, we established a comprehensive approach for characterizing micellar organization without relying on preassumed models. It is important to emphasize that the robustness of the extracted parameters does not arise from fitting to a specific geometric model, but from the internal mathematical consistency of the inversion framework across reciprocal and real space.

Analysis of the structure factor *S*(*Q*) revealed that intermicellar interactions are governed by screened Coulomb potentials, with their strength and range determined by both the number and spatial distribution of ionic residues along the hydrophilic block. Positioning ionic residues near the hydrophobic/hydrophilic block junction increases the range of electrostatic repulsion, while split-charge motifs produce stronger, longer-ranged repulsion than block-charge counterparts when charge residues are equivalently positioned along the chain (i.e., terminal, medial or junctional position), highlighting the key roles of charge delocalization in modulating intermicellar interactions.

Reconstructed excess scattering length density profiles Δρ­(*r*) provided direct real-space measures of intramicellar structure. The extracted micellar radius (*R*), radius of gyration (*R*_g_), and aggregation number (*N*_agg_) revealed systematic trends with ionic monomer placement. Comparisons between the effective charge (*Z*) and *N*_agg_ highlighted the influence of invasive water within the micellar core, with the *Z*/(*N*_agg_ *n*_c_) ratio serving as a collective descriptor for both counterion association and water penetration. While all sequence-defined polypeptoid BCPs form core–shell-type micelles, single-charged micelles are larger with greater radial chain extension than triple-charged ones when charged residues are at equivalent positions along the chains (i.e., terminal, medial or junctional position). Furthermore, micellar size decreases and coronal chains become more compact as ionic residues are positioned closer to the hydrophobic/hydrophilic block junction, accompanied by increased water penetration into the micellar core. These trends in semidilute micellar solutions are consistent with those observed under dilute conditions.^56,58^ Additionally, split-charge motifs exhibit greater invasive water than block-charge counterparts, highlighting the significant influence of charge delocalization on the intramicellar structure.

Overall, this study demonstrates that precise sequence control offers a molecular handle for tuning micellar size, density distribution, and intermicellar interactions via charge patterning. The integration of advanced model-free, data-driven inversion methods with SANS establishes a versatile framework for probing soft matter systems with complex internal architectures. These insights provide design principles for leveraging sequence-defined polymers to regulate electrostatics-driven self-assembly in aqueous environments.

## Supplementary Material



## Notes

Synthesis of sequence-defined peptoid block copolymers, MALDI-TOF MS and HPLC characterizations, experimental and fitted form factors, and reconstructed modified excess SLD profiles of SEQ 1 – SEQ 9 micelles (PDF)

## ⊥

Department of Physics and Chemistry, Milwaukee School of Engineering, Milwaukee, WI 53202, United States
