# Supplementary material for: Programmable Electrostatics in Charge-Patterned Polypeptoid Micelles Probed by Small-Angle Neutron Scattering
Source: Macromolecules. 2026 Apr 24;59(9):5569–81. doi: 10.1021/acs.macromol.5c03433 (PMC13173646; doi:10.1021/acs.macromol.5c03433)
Supplement: Supplementary file 1 [file ma5c03433_si_001.pdf]

Supplementary Information

Programmable Electrostatics in Charge-Patterned Polypeptoid Micelles Probed by Small-Angle Neutron Scattering

Erin Tsai,[a] Chi-Huan Tung,[b] Bailee N. Barrett,[a,c] Guan-Rong Huang,[d,e] Chang-Woo Do,[b] Wei-Ren Chen[b] and Donghui Zhang [a,*]

[a]Department of Chemistry and Macromolecular Studies Group, Louisiana State University, Baton Rouge, LA 70803, United States

[b] Neutron Scattering Division, Oak Ridge National Laboratory, Oak Ridge, 37831, Tennessee, United States

[c] Current address: Department of Physics and Chemistry, Milwaukee School of Engineering, Milwaukee, WI 53202, United States.

[d] Department of Engineering and System Science, National Tsing Hua University, Hsinchu, 30013, Taiwan

[e] Physics Division, National Center for Theoretical Sciences, Taipei, 10617, Taiwan

Corresponds to: dhzhang@lsu.edu

1. Materials. All solvents were used as received unless otherwise noted. Acetic anhydride (Reagent grade), triethylamine (Reagent grade), trifluoroacetic acid (Reagent grade), tetrahydrofuran (Optima), methylene chloride (HPLC grade), *N*,*N*-dimethylformamide (DMF, ACS grade), methanol (Reagent grade), acetonitrile (Reagent grade), and *N*-methylpyrrolidone were all purchased from Fisher Scientific. Rink amide resin, bromoacetic acid (≥99%), and *N*,*N*'-diisopropylcarbodiimide (≥99%) were purchased from Chem Impex International, *n*-Decyl amine (>98%), 2-methoxyethylamine (≥98%), and 4-methylpiperidine (≥98%) were obtained from TCI. β-alanine *t*-butyl ester hydrochloride (95%) was purchased from Chem Impex International and sodium hydroxide pellets (ACS) were purchased from VWR Life Sciences. Deuterium oxide (≥99%) was purchased from Cambridge Lab Isotopes.

2. Synthesis of Sequence-Defined Polypeptoid Block Copolymers. Polypeptoid block copolymers were synthesized by the solid-phase sub-monomer method using an adapted procedure (**Scheme S1**) on a Prelude X Peptide Synthesizer.[S1-S4] In a representative synthesis, Rink amide resins (495 μmol $-NH_2$, 0.67 mmol/g) were deprotected in a DMF solution of 20% 4-methylpiperidine for 12 min. The resulting amino groups on the resins were acylated by reacting with bromoacetic acid in DMF (1 mL, 0.4 M) with 1 eq. of *N*,*N*'- diisopropylcarbodiimide (DIC) for 20 min. Subsequently the resins were washed with DMF (5 × 20mL). In the amine displacement step, the acylated resins were treated with the appropriate amine (*n*-decylamine, 2-methoxyethyl, or β-alanine *t*-butyl ester hydrochloride) in either DMF (2-methoxyethylamine and β-alanine *t*-butyl ester hydrochloride) or *N*-methylpyrrolidone (*n*-decylamine). A concentration of 2.67 M of amine was employed for the displacement step with *n*-decylamine and 2-methoxyethylamine and with a reaction time of 30 min. For the displacement step of β-alanine *t*-butyl ester hydrochloride, a concentration of 2.67 M of amine was used with the addition of triethylamine (6 mL, 3.6 M in DMF) and reacted for 90 min. For the displacement step of the β-alanine *t*-butyl ester hydrochloride, an additional treatment of β-alanine *t*-butyl ester hydrochloride with a concentration of 2.67 M with triethylamine (6 mL, 3.6 M in DMF) was added to the reaction vessel and reacted for an additional 90 min after the excess amine solution was collected. Upon completion of the displacement of alanine *t*-butyl ester hydrochloride, the resin was mixed with chloroform (5 mL), followed by subsequent resin washes with DMF (10 mL), chloroform (3 × 10 mL), and DMF (3 × 20 mL). For all other residue additions, the resin was simply washed with DMF (3 × 10 mL) after displacement. The resins were then repeatedly washed with DMF (5 × 20

mL). The acylation and displacement steps were repeated until the desired chain lengths were achieved. The peptoid-functionalized resins were then resuspended in DCM. The peptoid chains were cleaved and deprotected using 30% TFA in DCM for 40 mins. The solution was evaporated under nitrogen. Crude peptoid polymers (800 mg) dissolved in THF (12 mL) and TEA (2-3 mL) were allowed to react with acetic anhydride (600 mL) at room temperature for 2-3 hours to acylate the *N*-terminus of chains. The solution was evaporated under nitrogen. The resulting peptoid oligomers were redissolved in deionized water and purified by dialysis against DI water for 2 days. The resulting solutions of peptoid polymer were lyophilized to produce a white, fluffy powder.

Scheme S1. Representative synthesis of SEQ 1 by the sub-monomer method.

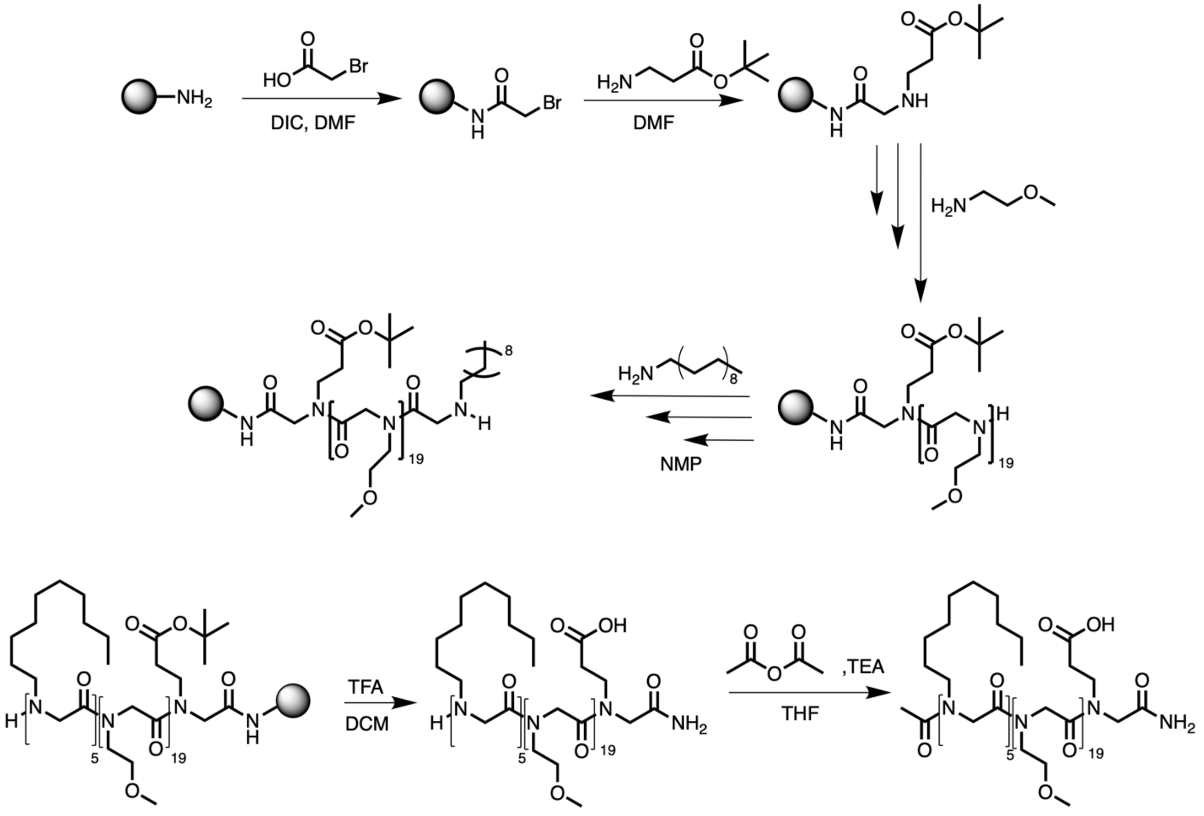

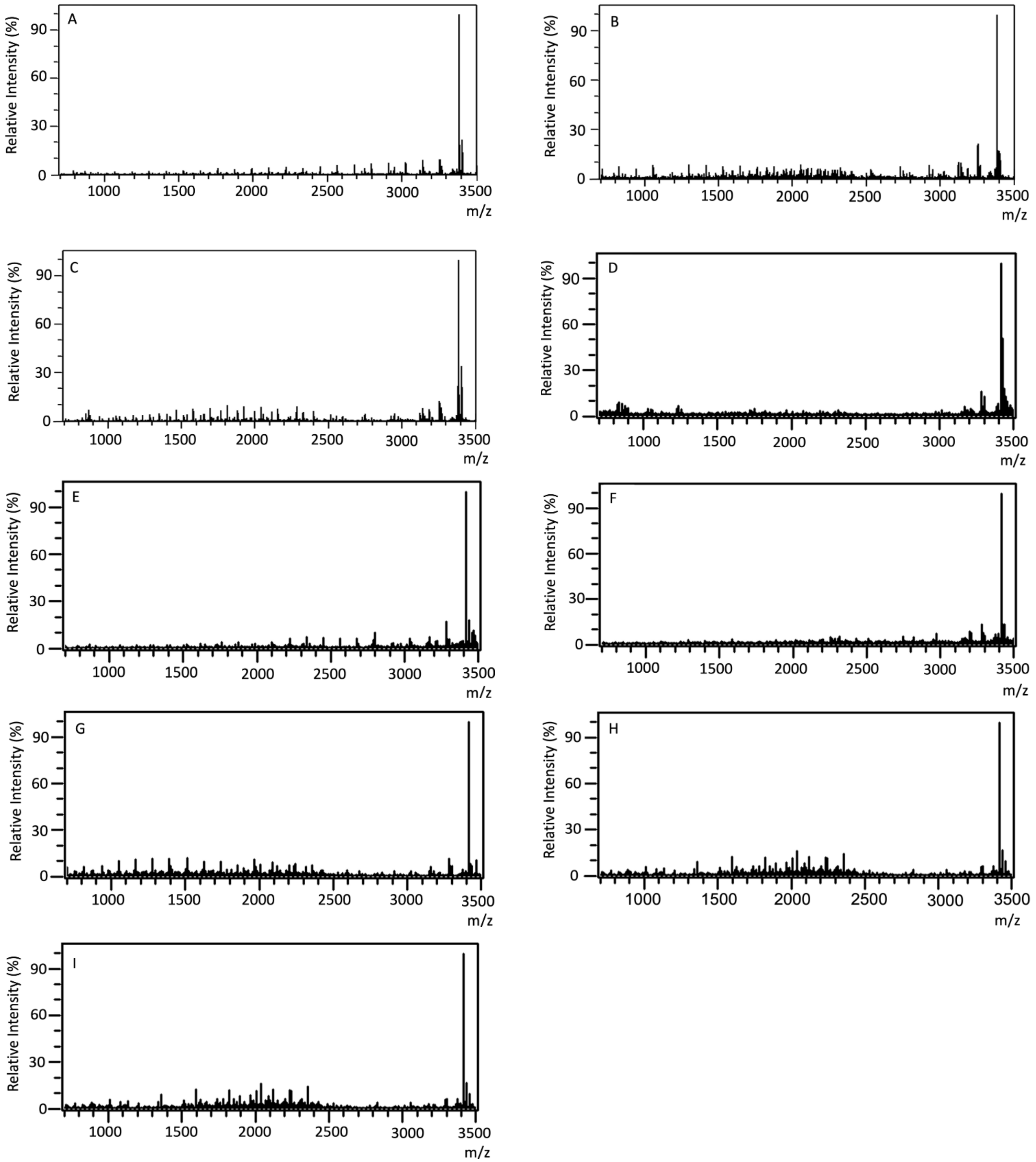

Fig. S1. (A-I) Representative MALDI-TOF spectra for sequence-defined polypeptoid block copolymers (SEQ 1 - SEQ 9).

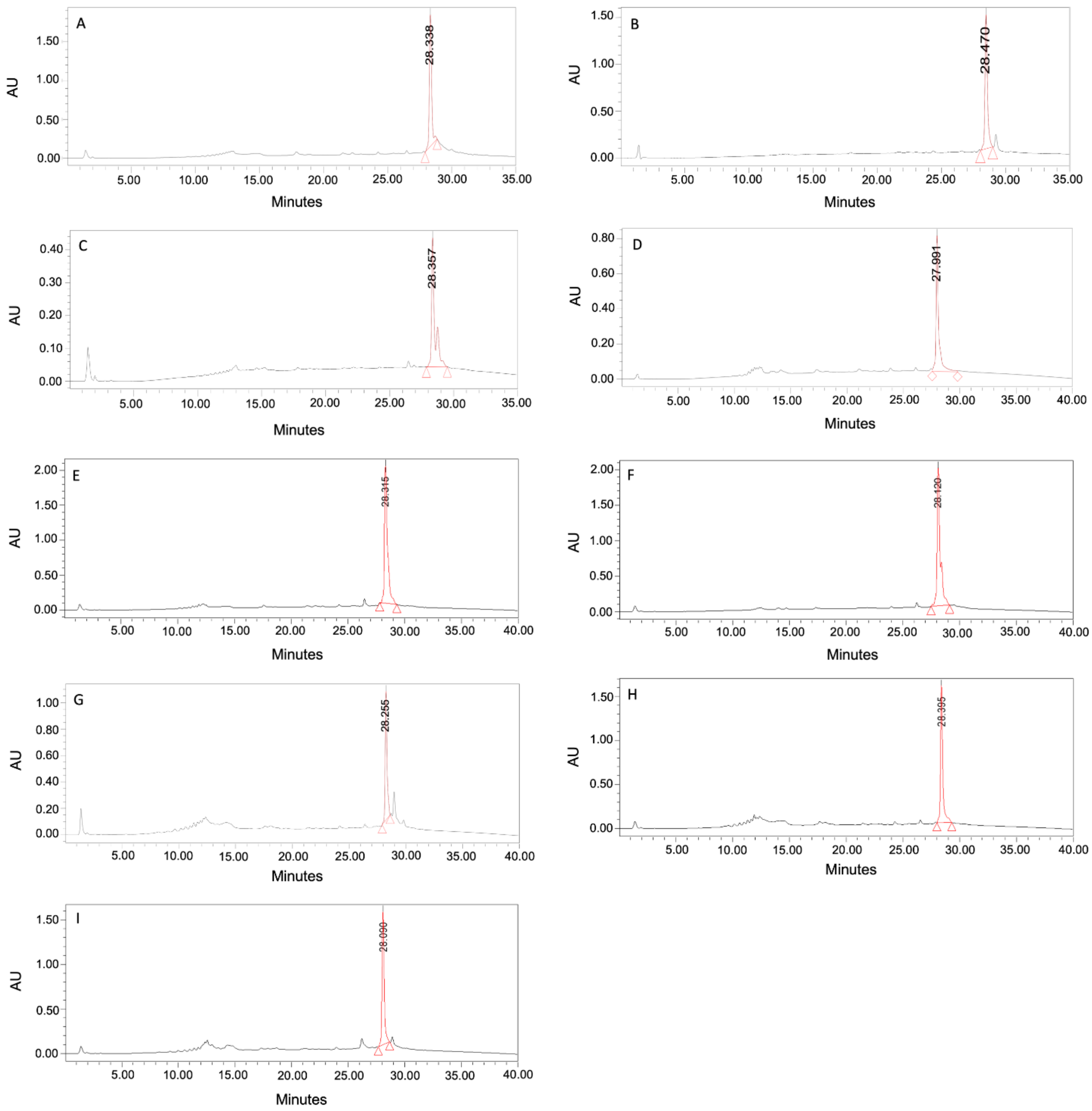

Fig. S2. (A-I) Representative HPLC chromatograms for sequence-defined polypeptoid block copolymers (SEQ 1 – SEQ 9).

Table S1. Experimentally determined exact molecular weights of sequence-defined polypeptoid block copolymers (SEQ 1 - SEQ 9) by MALDI-TOF MS analysis, the calculated exact molecular weights using the molecular formula, and the sample purity determined by RP-HPLC analysis.

Sequence	Molecular Formula		Calc. (m/z)	Found (m/z)	Purity (%)
SEQ 1	$C_{162}H_{298}N_{26}O_{47}Na^+$<br>$C_{162}H_{297}N_{26}O_{47}Na_2^+$	[M+Na] $^+$<br>$[M+2Na-H]^+$	3383.16<br>3404.15	3383.03<br>3405.02	90%
SEQ 2	$C_{162}H_{298}N_{26}O_{47}Na^+$<br>$C_{162}H_{298}N_{26}O_{47}K^+$<br>$C_{162}H_{297}N_{26}O_{47}Na_2^+$	$[M+Na]^+$<br>$[M+K]^+$<br>$[M+2Na-H]^+$	3383.16<br>3399.14<br>3404.15	3383.13<br>3399.10<br>3404.11	96%
SEQ 3	$C_{162}H_{298}N_{26}O_{47}Na^+$<br>$C_{162}H_{298}N_{26}O_{47}K^+$<br>$C_{162}H_{297}N_{26}O_{47}Na_2^+$	$[M+Na]^+$<br>$[M+K]^+$<br>$[M+2Na-2H]^+$	3383.16<br>3399.14<br>3404.15	3381.19<br>3398.16<br>3404.16	96%
SEQ 4	$C_{162}H_{294}N_{26}O_{49}Na^+$<br>$C_{162}H_{293}N_{26}O_{49}Na_2^+$<br>$C_{162}H_{292}N_{26}O_{49}Na_3^+$<br>$C_{162}H_{293}N_{26}O_{49}K_2^+$	$[M+Na]^+$<br>$[M+2Na-H]^+$<br>$[M+3Na-2H]^+$<br>$[M+2K-H]^+$	3411.12<br>3433.10<br>3455.08<br>3665.04	3410.37<br>3432.35<br>3454.32<br>3466.41	89%
SEQ 5	$C_{162}H_{294}N_{26}O_{49}Na^+$<br>$C_{162}H_{294}N_{26}O_{49}K^+$<br>$C_{162}H_{293}N_{26}O_{49}NaK^+$<br>$C_{162}H_{293}N_{26}O_{49}K_2^+$	$[M+Na]^+$<br>$[M+K]^+$<br>$[M+Na+K-H]^+$<br>$[M+2K-H]^+$	3411.12<br>3427.09<br>3449.07<br>3665.04	3410.62<br>3426.59<br>3448.55<br>3465.54	94%
SEQ 6	$C_{162}H_{294}N_{26}O_{49}Na^+$<br>$C_{162}H_{294}N_{26}O_{49}K^+$<br>$C_{162}H_{293}N_{26}O_{49}Na_2^+$<br>$C_{162}H_{293}N_{26}O_{49}NaK^+$	$[M+Na]^+$<br>$[M+K]^+$<br>$[M+2Na-H]^+$<br>$[M+Na+K-H]^+$	3411.12<br>3427.09<br>3433.10<br>3449.07	3410.48<br>3426.45<br>3432.43<br>3448.41	95%
SEQ 7	$C_{162}H_{294}N_{26}O_{49}Na^+$<br>$C_{162}H_{294}N_{26}O_{49}K^+$<br>$C_{162}H_{293}N_{26}O_{49}Na_2^+$<br>$C_{162}H_{293}N_{26}O_{49}K_2^+$	$[M+Na]^+$<br>$[M+K]^+$<br>$[M+2Na-H]^+$<br>$[M+2K-H]^+$	3411.12<br>3427.09<br>3433.10<br>3665.04	3410.41<br>3425.38<br>3432.38<br>3466.40	80%
SEQ 8	$C_{162}H_{294}N_{26}O_{49}Na^+$<br>$C_{162}H_{293}N_{26}O_{49}Na_2^+$<br>$C_{162}H_{292}N_{26}O_{49}Na_3^+$	$[M+Na]^+$<br>$[M+2Na-H]^+$<br>$M+3Na-2H]^+$	3411.12<br>3433.10<br>3455.08	3410.43<br>3432.41<br>3454.38	84%
SEQ 9	$C_{162}H_{294}N_{26}O_{49}Na^+$<br>$C_{162}H_{294}N_{26}O_{49}K^+$<br>$C_{162}H_{293}N_{26}O_{49}Na_2^+$	$[M+Na]^+$<br>$[M+K]^+$<br>$[M+2Na-H]^+$	3411.12<br>3427.09<br>3433.10	3410.47<br>3426.43<br>3431.44	83%

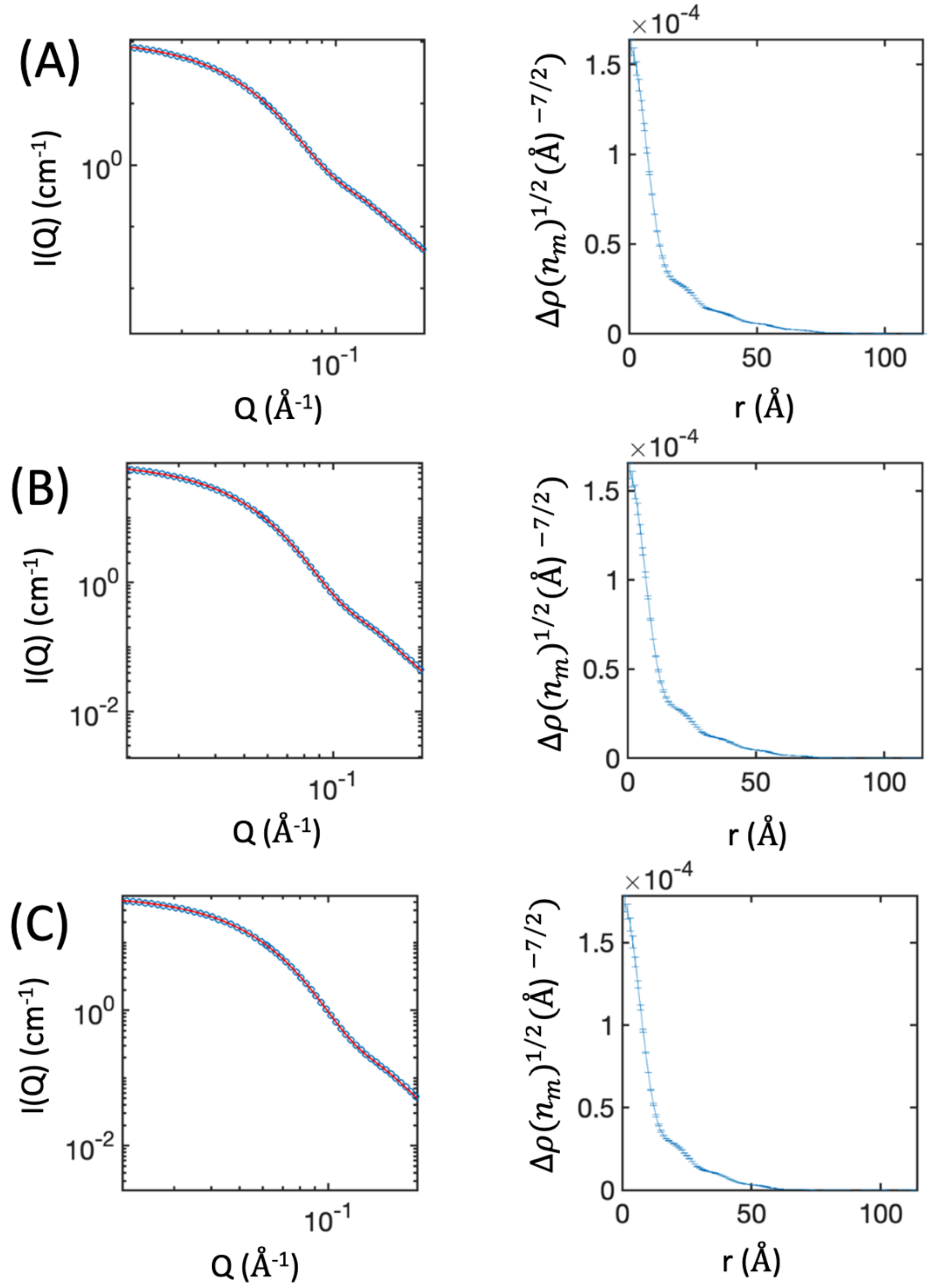

Fig. S3. (A-C) *Left*: Experimental (blue circles) and fitted form factor *P(Q)* (red line) of SEQ 1 – SEQ 3 (single charge) micelles at 100% D_2O contrast, used for phase retrieval. *Right:* Reconstructed modified excess SLD profile $\Delta\rho(r)(n_m)^{1/2}$, obtained using a PhaseLift algorithm.

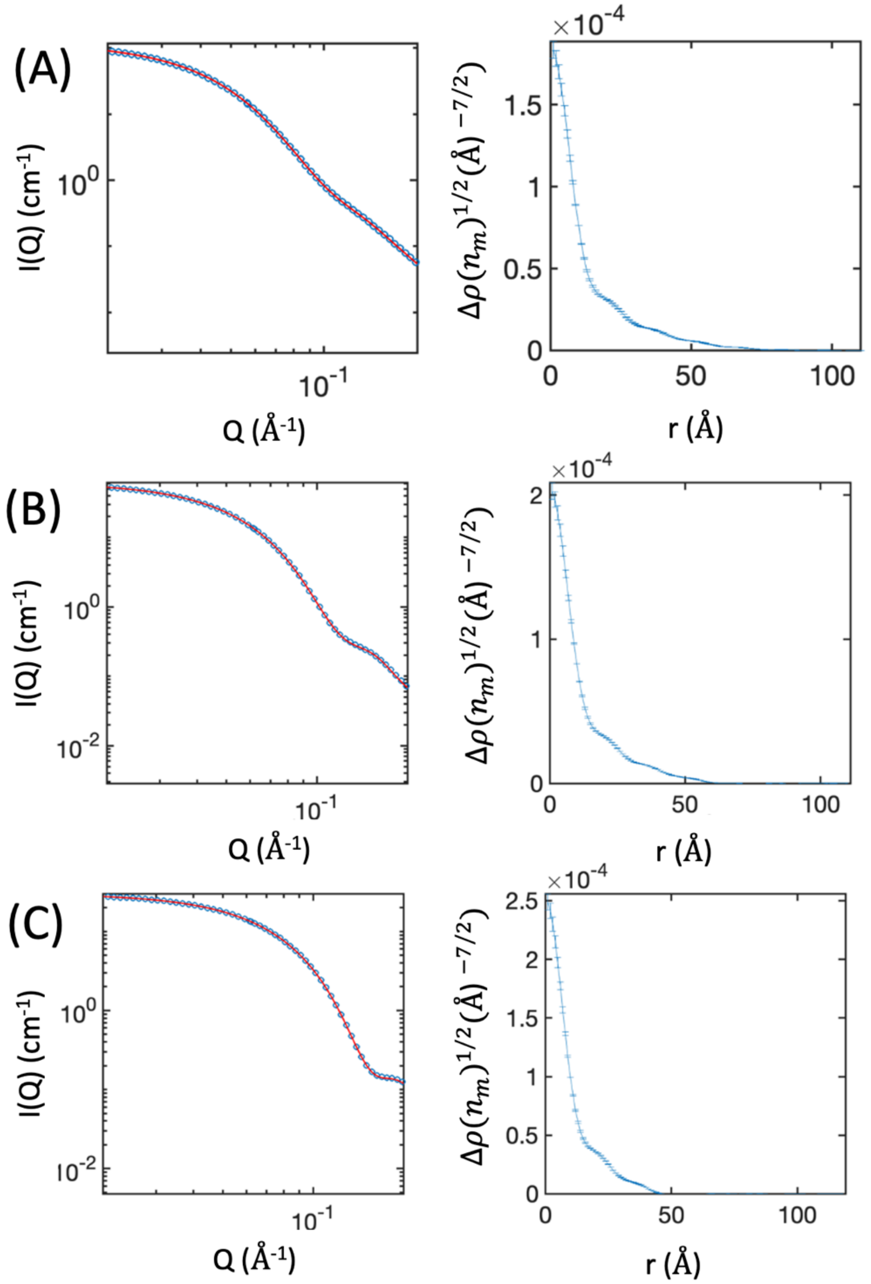

Fig. S4. (A-C) *Left*: Experimental (blue circles) and fitted form factor *P(Q)* (red line) of SEQ 4 – SEQ 6 (triple charges, split) micelles at 100% D_2O contrast, used for phase retrieval. *Right:* Reconstructed modified excess SLD profile $\Delta\rho(r)(n_m)^{1/2}$, obtained using a PhaseLift algorithm.

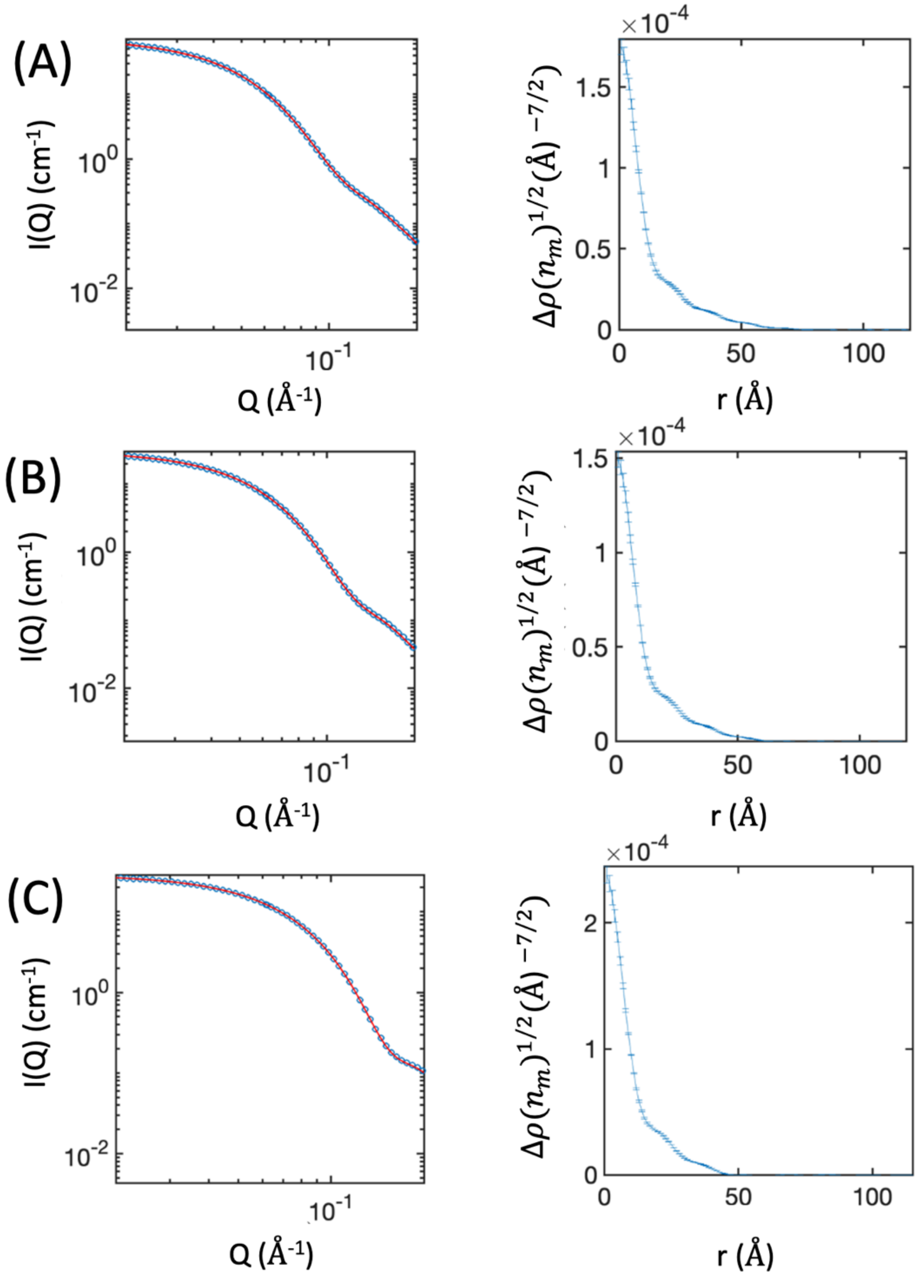

Fig. S5. (A-C) *Left*: Experimental (blue circles) and fitted form factor *P(Q)* (red line) of SEQ 7 – SEQ 9 (triple charges, block) micelles at 100% D_2O contrast, used for phase retrieval. *Right:* Reconstructed modified excess SLD profile $\Delta\rho(r)(n_m)^{1/2}$, obtained using a PhaseLift algorithm.

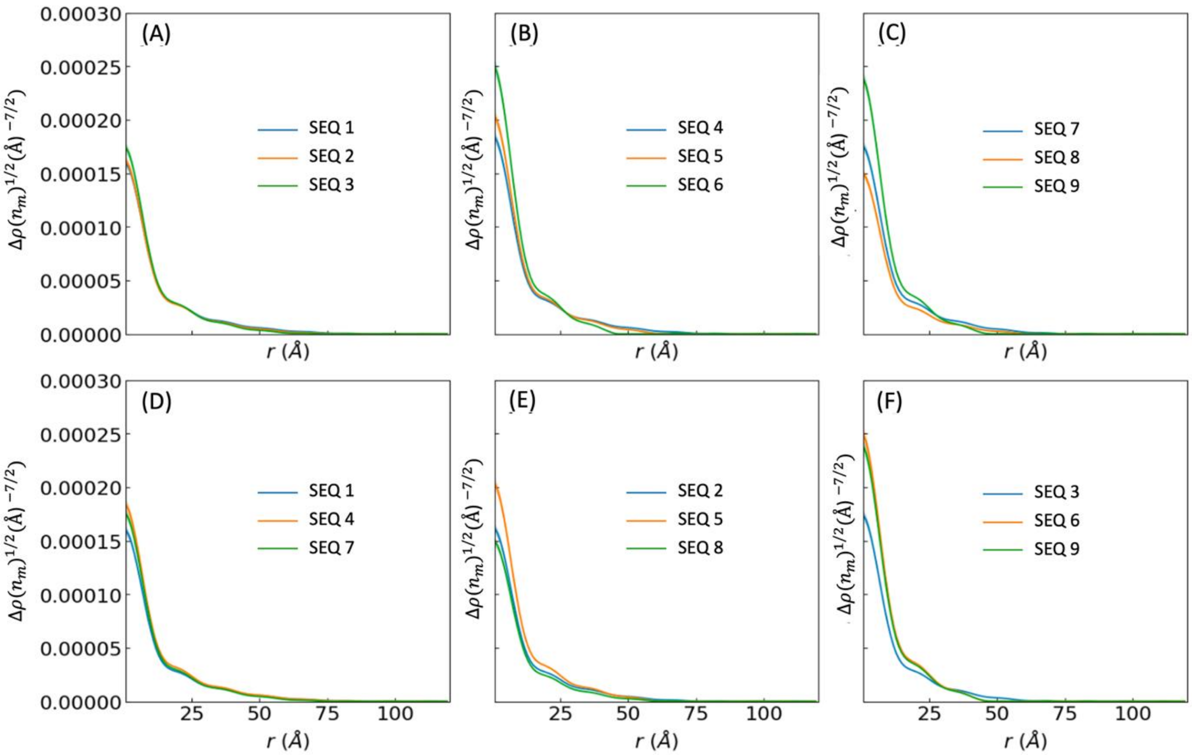

Fig. S6. Reconstructed modified excess SLD profiles, $\Delta\rho(r)(n_m)^{1/2}$, for SEQ 1 – SEQ 9 obtained using orthonormal basis expansion and PhaseLift.

References

S1. Zuckermann, R. N.; Kerr, J. M.; Kent, S. B. H.; Moos, W. H. Efficient method for the preparation of peptoids [oligo(N-substituted glycines)] by submonomer solid-phase synthesis. *J. Am. Chem. Soc.* **1992**, *114*, 10646−10647.

S2. Connolly, M. D.; Xuan, S.; Molchanova, N.; Zuckermann, R. N. Chapter Eight - Submonomer synthesis of sequence defined peptoids with diverse side-chains. In *Methods in Enzymology*; Petersson, E. J., Ed.; Academic Press, **2021**.

S3. Sternhagen, G. L.; Gupta, S.; Zhang, Y.; John, V.; Schneider, G. J.; Zhang, D. Solution self-assemblies of sequence-defined ionic peptoid block copolymers. *J. Am. Chem. Soc.* **2018**, *140*, 4100−4109.

S4. Barrett, B. N.; Tung, C. H.; Huang, G. R.; Hossain, I.; Do, C. W.; John, V. T.; Chen, W. R.; Zhang, D. Modulating Water Distribution and the Intramicellar Assembly of Sequence-Defined Ionic Peptoid Block Copolymers by the Ionic Monomer Position. *Macromolecules* **2023**, *56*, 5306−5313.
